# Redo-EVAR After Surgical Repair in Ruptured Abdominal Aortic Aneurysm

**DOI:** 10.15171/jcvtr.2015.38

**Published:** 2015-12-01

**Authors:** Şahin Bozok, Sedat Ozan Karakişi, Şaban Ergene, Nebiye Tufekçi, Gökhan Ilhan, Hakan Karamustafa

**Affiliations:** Department of Cardiovascular Surgery, Recep Tayyip Erdogan University Faculty of Medicine, Rize, Turkey

**Keywords:** Endovascular Aneurysm, EVAR, Abdominal Aorta, Aneurysm

## Abstract

Endovascular aneurysm repair (EVAR) is an adequate means for treating infrarenal abdominal aortic aneurysms (AAA). However, secondary interventions are required in approximately 15% to 20% of patients. The aim of this paper was to report our knowledge with stent grafts in secondary interventions after EVAR in a 73-year-old patient. One of the exceptional complications of EVAR are endoleaks which may lead to expansion of aneurysm and rupture if not repaired.

## Introduction

Endovascular aneurysm repair (EVAR) is an acceptable means for treating infrarenal abdominal aortic aneurysms (AAA). However, complications of endoleaks such as migration of stent graft, fracture of stent, dilatation of the aortic neck) or occurrence of further aortoiliac aneurysms necessitate long-term follow-up of the patient, and secondary interventions are required in approximately 15% to 20% of patients.^1-3^ Recent advances in stent graft technology has certainly led to routine use of EVAR in favourable as well as unfavourable circumstances.

The aim of the present report was to report our knowledge with stent grafts in secondary interventions after EVAR in a 73-year-old patient.

## Case Report

**Figure 1:**
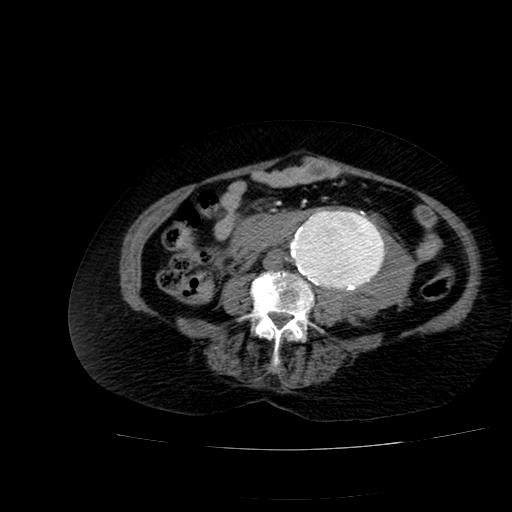
Computed tomography showing ruptured infrarenal aortic aneurysm.

**Figure 2:**
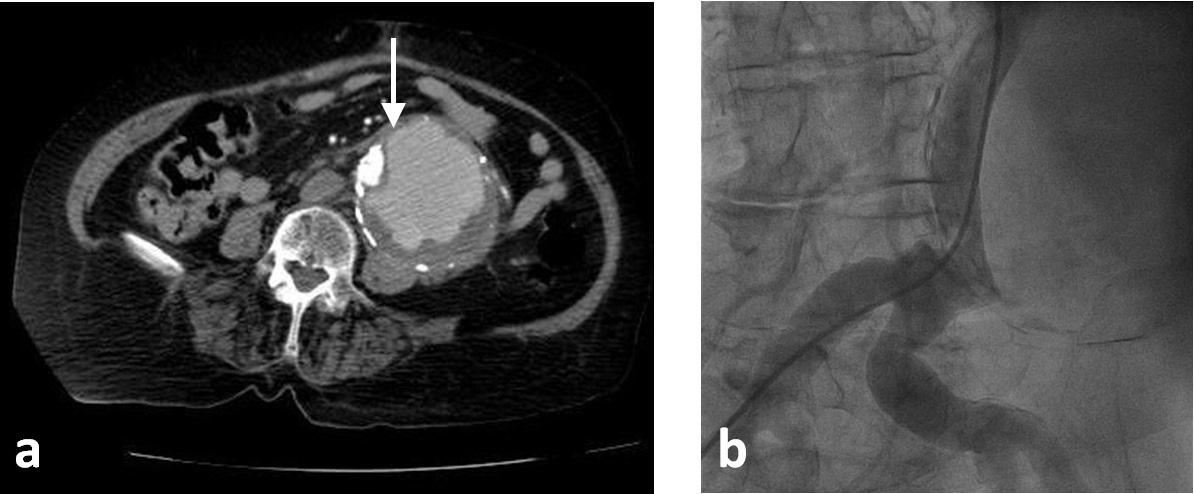
Control computed tomography showing extravasation of contrast (white arrow) into the aneurysmal sac.

**Figure 3:**
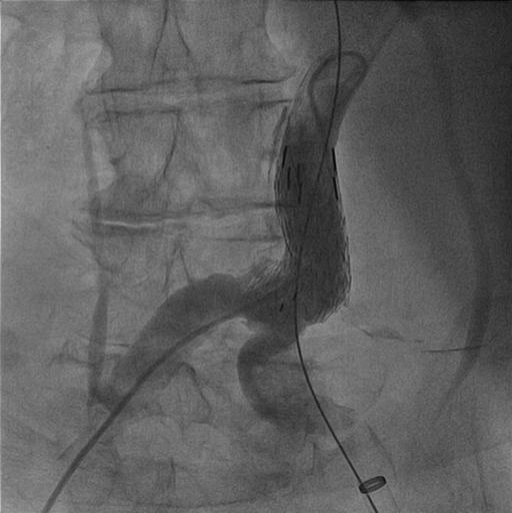
Angiography showing leakage from posterior anastomosis line of graft and successfully treated with GORE EXCLUDER AAA Endoprosthesis stent graft.

**Figure 4:**
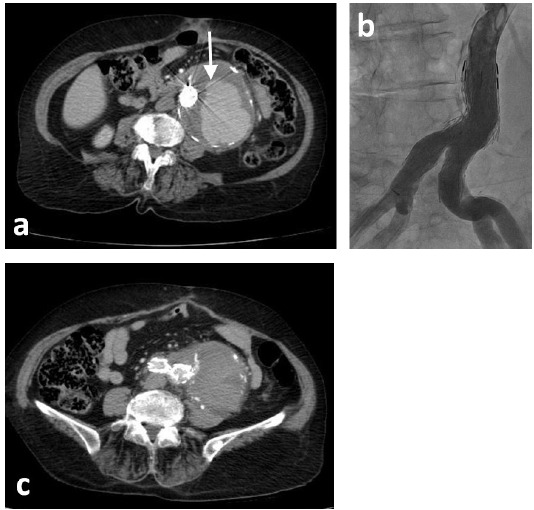
Type 1b endoleak from distal of the aortic extension graft to the aneurysm sac in follow up computed tomography obtained one month after endovascular repair (white arrow) (a). A bifurcated stent graft was successfully implanted into the old aortic extension graft (b). The postoperative computed tomography showing complete exclusion of the aneurysm with no leakage (c).

**Figure 5:**
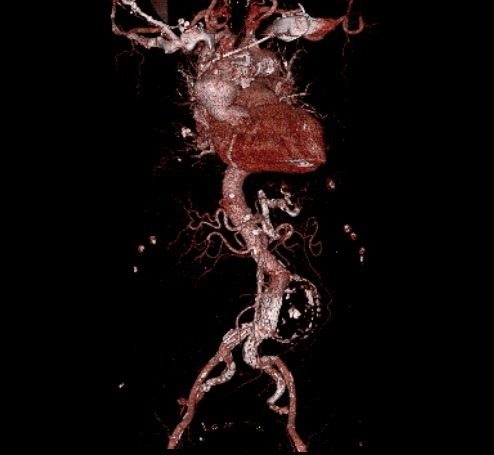
The postoperative computed tomography angiography showing complete exclusion of the aneurysm with no leakage.

A 73-year-old woman who was receiving treatment for hypertension and aortic valve replacement, admitted to emergency service with hypotension and abdominal pain. Computed tomography (CT) showed ruptured infrarenal aortic aneurysm and the patient was successfully operated with 28 mm Dacron tube graft (Figure 1). Three months later, the patient admitted with abdominal pain again. Control CT scan with contrast performed in the emergency room revealed contrast extravasation into the aneurysmal sac (Figure 2a, b). After angiography, we detected a leakage from posterior anastomosis line of graft and successfully treated with GORE EXCLUDER AAA Endoprosthesis stent graft (WL Gore and Associates, Flagstaff, AZ) (Figure 3). In follow up CT obtained one month after endovascular repair, we detected type 1b endoleak from distal of the aortic extension graft to the aneurysm sac (Figure 4a). A bifurcated stent graft (Endologix, IntuiTrak® Delivery System, Irvine, California, USA) was effectively implanted into the old aortic extension graft and the middle portion of the right common iliac artery; the contralateral iliac limb was introduced percutaneously (Figure 4b). The postoperative follow-up was at our facility and was uneventful. On the seventhpostoperative day and first month, a postoperative CT scan was performed and revealed complete aneurysm recovery without any leakage (Figure 4c, Figure 5).

## Discussion

The fundamental goal of AAA repair by surgical or endovascular means is to reduce the risks for aneurysm rupture and death. Today, EVAR is considered relatively safe and effective for treatment of infrarenal AAA and is often considered as the first choice therapy in patients with favourable aortoiliac morphology. However, none of the currently available devices are completely effective in preventing aneurysm rupture after EVAR, and lifelong surveillance of these stent grafts and aneurysms is needed. When secondary interventions are required for EVAR failure, there are a variety of reasons and means of treatment that depend not just on the accepted standards but also on local expertise in dealing with the stent graft failures.^3-6^

Patients presenting with stent graft failure after EVAR tend not to exhibit profound signs of hemodynamic collapse, and their symptoms are generally that of abdominal and back pain.^7^ Detailed evaluation to identify the aetiology of stent graft failure and aneurysm rupture is vital for planning for redo-EVAR. In the present case, control CT scan with contrast performed on emergency basis, which documented the extravasation of contrast into the aneurysmal sac.

The most common adverse factors contributing to aneurysm rupture after EVAR included type I endoleak with stent graft migration (63%), type II endoleak (19%), type I endoleak without stent graft migration (11%), and in 7% of patients, the aetiology for aneurysm rupture after EVAR was undetermined.^8^ Treatment options were designed on the basis of underlying aetiology. The presence of type I endoleak with or without stent graft migration was a significant risk factor for AAA rupture; it is our routine practice to treat any type I endoleak at the time of diagnosis. In the present case, follow up CT detected type 1b endoleak from distal of the aortic extension graft to the aneurysm sac and treated successfully.

Our routine postoperative EVAR surveillance includes clinical evaluation and duplex ultrasound at 1 month and every 6 months, as well as a CT angiography evaluation at 1 month, 6 months, 12 months, and yearly thereafter. In the present case, follow up CT obtained one month after EVAR revealed endoleak incidentally.

One of the exceptional complications of EVAR are endoleaks which may lead to expansion of aneurysm and rupture if not repaired. In narrow distal aorta as in our case, we can use bifurcated grafts to prevent the endoleak and save time.

## Ethical Issues

None to be declared.

## Competing Interests

The authors declared no conflicts of interest with respect to the authorship and/or publication of this article.
